# Embodying the avatar of an omnipotent agent modulates the perception of one’s own abilities and enhances feelings of invulnerability

**DOI:** 10.1038/s41598-022-26016-1

**Published:** 2022-12-14

**Authors:** Althea Frisanco, Michael Schepisi, Gaetano Tieri, Salvatore Maria Aglioti

**Affiliations:** 1 Fondazione Istituto Italiano Di Tecnologia (IIT), Sapienza University of Rome and Center for Life Nano- & Neuroscience, Rome, Italy; 2 IRCCS Santa Lucia Foundation, Rome, Italy; 3 Virtual Reality Lab, Unitelma Sapienza University, Rome, Italy

**Keywords:** Neuroscience, Psychology

## Abstract

Immersive virtual reality can give people the illusion of owning artificial bodies (i.e., avatars) and controlling their actions. Tellingly, people appear to adhere to the newly embodied entities not just on the basis of physical traits but also behaving accordingly with the hallmarks of the represented characters. In two studies we pushed the limits of this process by testing if one’s own sense of power could be affected by embodying the anthropomorphic representation of the Christian God, that is considered an omnipotent entity. A human Muscled and a Normotype avatar were used as controls. Results showed that participants embodying the God-avatar: (i) reacted to a threatening event compromising their physical safety by exhibiting a lower skin conductance response and heart rate deceleration compared to the Normotype-control avatar (Study 1); (ii) estimated they had more physical abilities compared to both the Normotype-control and the Muscled-control avatars (Study 2). Taken together, our findings suggest that embodying an omnipotent agent may exert an influence on people’s perception of their own limits and capabilities, nourishing feelings of physical invulnerability and strength. Our study indicates that effectively embodying virtual role models may boost achievements and have translational implications in the field of empowerment.

## Introduction

The last decades have witnessed the growth of the literature on body ownership illusion, namely the perceptual illusion that a fake body part is part of one’s own body^1^. Starting from the widely-known paradigm of the rubber hand illusion (RHI^2^), more and more attention has been given to the investigation of the body representation and the extraordinary plasticity of our brain.

State-of-the-art tools, like immersive virtual reality (IVR), allowed researchers to take a further step: in addition to reproducing classical paradigms like RHI, by replacing the real hand with a virtual limb which people actually felt as their own^3–10^, researchers have demonstrated that the illusion can work also at the whole body level^11–14^.

Specifically, the illusion to own a whole virtual body (i.e., *full body illusion or virtual embodiment*) is given when people are immersed in a virtual environment through a head mounted display (HMD) and see the virtual body from first-person perspective (1PP) located in the exact spatial position of their own real body. Although the virtual embodiment is present even in the absence of motions (or even when virtual movements do not match real ones^15,16^), there is agreement that the illusion can be further enhanced by making the virtual body synchronously move with the participant’s real movements^17–20^, or by making the participants observe the virtual body touched while simultaneously feeling the touch on corresponding real body areas^14,21–23^. Moreover, recent technological advancements opened novel ways to design fully detailed avatars to be embodied, making the virtual bodies more and more realistic, and allowing researchers to study the implications of getting specific physical connotations. In fact, attributes that characterize virtual bodies have been shown to considerably influence people’s perceptions, attitudes and behaviours, a tendency referred to as “Proteus effect^24^”. Specifically, in their seminal work the authors observed that embodying an attractive or a tall avatar elicited, respectively, more intimate attitudes during social interactions and more dominant behaviours during a negotiation task as compared to an unattractive or smaller avatar.

Although researchers are still debating on the mechanisms which may drive this effect, a commonly accepted explanation states that people might feel de-individuated in the virtual environments where they are immersed without one’ own body and, in turn, they may adhere to a new identity that is inferred from the avatar they are embodying. In other words, the avatar becomes a primary identity cue, capable of reshaping one’s own self representation^24^.

Following the Yee and Bailenson’s work^24^, important studies have documented the transformative effects of embodying different virtual bodies, sensing the potential positive outcomes at both individual and social level. For instance, white people embodying a dark-skinned avatar have shown to decrease their implicit racial bias^17,25^, and offenders embodying a female victim of domestic abuse improved their ability to recognize fearful female faces, reducing their tendency to code them as happy^18^. Therefore, over time, IVR has been established to be a valuable tool for observing the role of relevant physical attributes in driving different behaviours (e.g.,^11,26–29^). Furthermore, by using advanced modelling software some authors have investigated the effects of virtually being in someone else’s shoes by creating avatars resembling certain characters. Two very exemplary studies have involved avatars resembling Albert Einstein and Sigmund Freud. The former, i.e., a character strongly associated with a great intelligence, led people to improve their cognitive task performances, suggesting that the embodiment illusion may be used to enhance executive functioning^30^. The latter, i.e., possibly the most famous counsellor ever, positively influenced the mood of the participants and allowed them to elaborate a more satisfactory solution to their personal problems, showing the power of the embodiment illusion to effect cognitive changes^31^.

Considering the growing and promising results in this research field and, particularly, the implications of using avatars resembling role models, here we aimed at testing the boundaries of the embodiment illusion by investigating the behavioural and physiological effects of embodying an abstract supernatural entity, i.e., the Christian God in its most commonly shared anthropomorphic representation (see Materials and Methods section for a description). Specifically, we explored whether virtually being in the shoes of God may affect one’s own sense of power, nourishing both feelings of physical invulnerability and grandiosity. Indeed, omnipotence is a trait often investigated by researchers exploring the representation of God who in the Christian religion is depicted as an ultimate, limitless and omnipotent agent^32^. For instance, a survey conducted by the Pew Research Center^33^ about beliefs concerning God revealed that 86% of the people who believe in God as described in the Bible thought he was omnipotent. Generally, omnipotence refers to the absolute power that does not meet obstacles^34^. This definition involves many facets and meanings, such as immortality or ability to perform an infinite number of tasks, that make it wide and vague. Moreover, we acknowledge that there is no unique way to conceptualize omnipotence, and it might be expressed in many distinct aspects. We considered two specific facets of omnipotence: in Study 1 we considered omnipotence in terms of invulnerability (i.e., not being harmed or injured) by measuring if embodying the God-avatar would decrease participants’ perception of feeling physically in danger. In Study 2 we considered omnipotence in terms of powerfulness, reflected in an increase of personal agency. In so doing, we measured if embodying the God-avatar would enhance participants’ estimation of their own physical abilities.

## Common methods

### Participants

Fifty-four healthy participants with normal or corrected to normal vision were recruited in both Study 1 and Study 2 (26 males; age: M = 25.46, SE = 5.98). Sample size was determined by means of priori power analyses: the number of subjects required for Study 1 was N = 42, considering a medium-small effect size of f = 0.20^35^, an α = 0.05 and a statistical power of 80% in a within-groups design with a single factor *Avatar* with 3 levels (God-avatar, Muscled-control avatar, Normotype-control avatar). To compensate for loss of participants due to technical and other issues, we recruited more participants. The final sample of fifty-four participants fitted also the number required for Study 2 (N = 32), considering the same effect size, α, and statistical power in a within-groups design with 3 *Avatar* within subjects × 3 *Depth* (low, medium, high) within-subjects ANOVA.

We recruited Italian participants to be sure that they shared the same Christian culture and thus the God-avatar recognition was comparable. We included Catholic-Christian, agnostic and atheist participants. Moreover, since the existence of a shared physical representation of God seems to be partially affected by its own physical features, like skin colour^36^, all participants were Caucasians. Exclusion criteria consisted of epilepsy, use of medication, recent consumption of alcohol, intellectual disability, mental health difficulties (e.g., requiring medication), and suffering from vertigo.

### Common experimental stimuli and setup

#### Avatars

**Figure 1:**
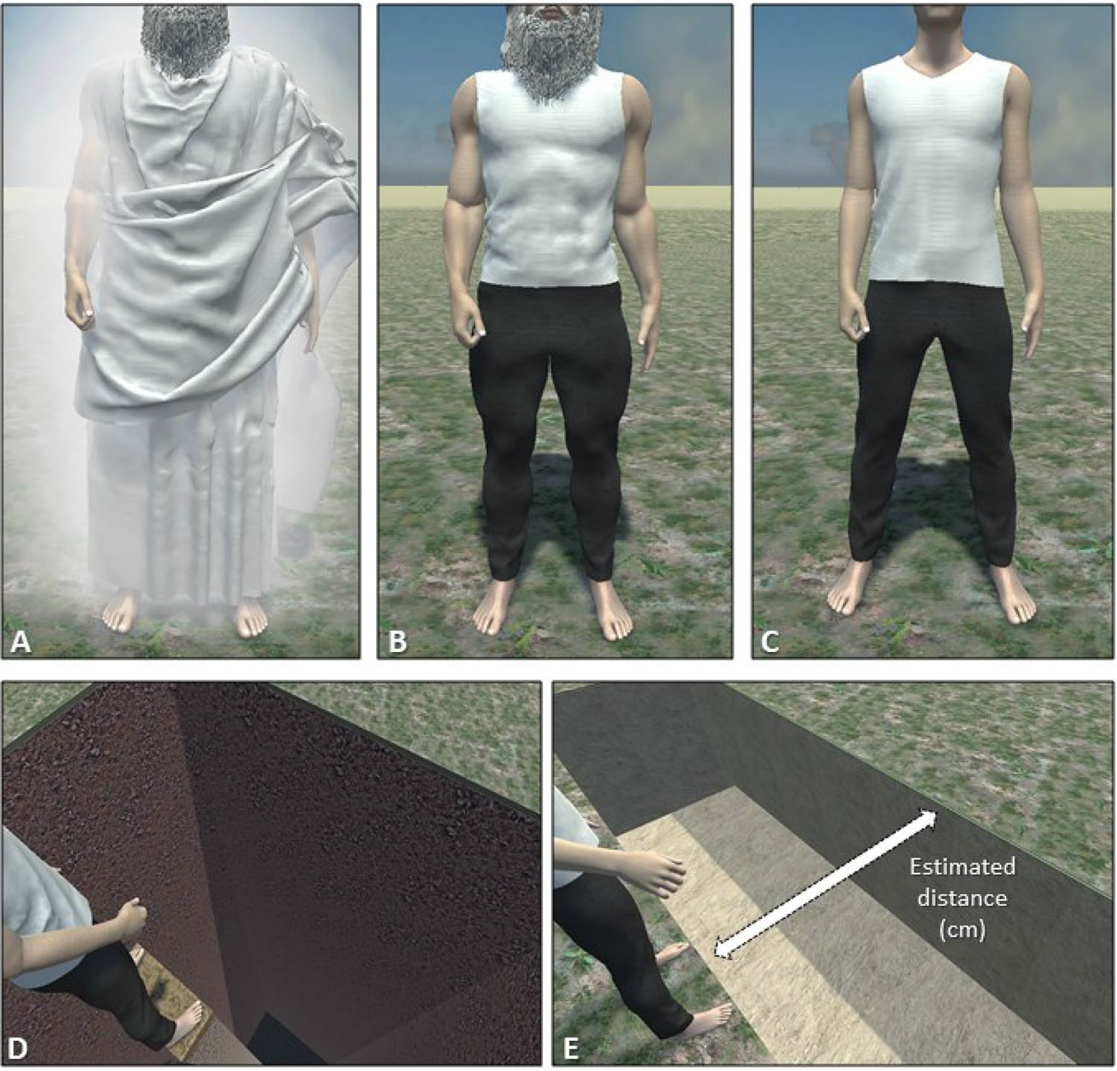
A schematic representation of the avatars and the experimental tasks. Upper part represents (**A**) God-avatar, (**B**) Muscled-control avatar, (**C**) Normotype-control avatar. Lower part shows an example of (**D**) threatening event (ground collapsing, Study 1) and (**E**) estimated distance task (moving ground, Study 2).

The three avatars used in both Study 1 and Study 2, were created by using Daz Studio (https://www.daz3d.com/), Marvelous Designer (https://www.marvelousdesigner.com/) and Autodesk 3D Studio Max 2019 (Autodesk Inc.), implemented in Unity Game engine software (Unity Technology, version 2018.3) and presented through the head-mounted display Oculus Rift (HMD; https://www.oculus.com/), having 110◦ field-of-view (diagonal FOV) with a resolution of 2160 × 1200. To design the avatar resembling God (Fig. 1A), we capitalized on the physical representation that people belonging to the Christian cultures seem to hold, and that art, literature and media contributed to widespread and fortify. According to this representation, God is depicted as an old white man, with a long beard and a long white dress^36,37^. We based the design of the God-avatar on Michelangelo’s painting “Creation of the Sun and Moon” (1508–1512), which has been shown to be recognized as God’s representation in past research^38^. To make sure that the avatar was recognizable, we run a pilot online survey where we presented a picture of the God-avatar and asked people to write what or who they thought was being shown. The answer “God” was the one given most frequently, with a high difference in frequency from the other answers (41.8 percentage points higher than the second most listed option, see Supplementary materials).

Moreover, two human avatars used as control were implemented. Since we designed the avatar of God as muscular and given the importance of this physical feature for the tasks that we administered, we decided to include an avatar with the same physical size. Thus, the first human avatar, which we will refer to as the Muscled-control avatar, had the same muscle mass of the God-avatar, but wore different clothing and was not provided with aura (Fig. 1B). The pilot study results showed that, despite the very same body mass, the Muscled-control avatar was rated as significantly more muscular (see Supplementary materials). The second, which we will refer to as Normotype-control avatar, had an ordinary male body, wore the same clothing as the Muscled-control avatar, and was not provided with aura (Fig. 1C).

**Figure 2:**
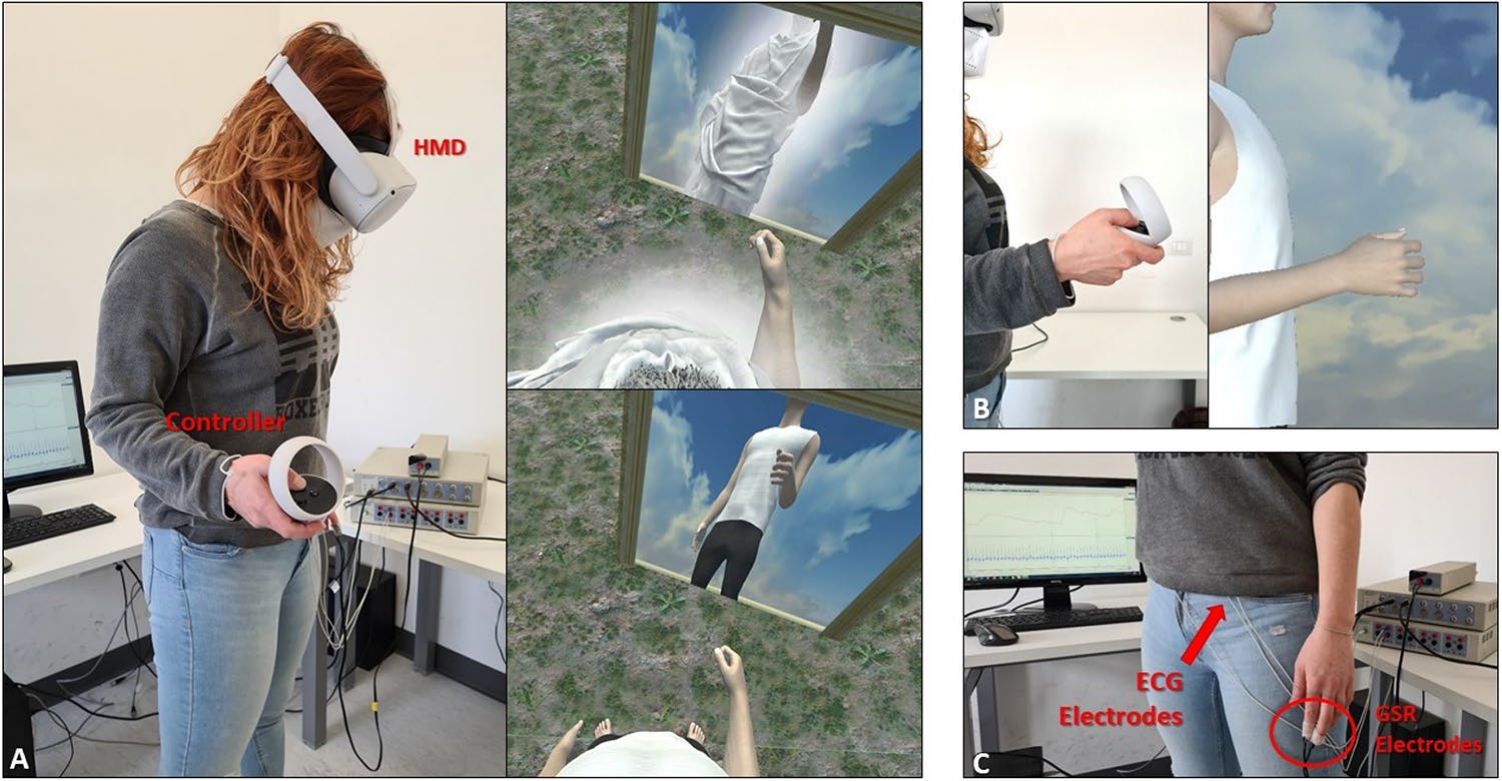
A schematic representation of the experimental setup. (**A**) The participant observing from 1PP the God-avatar (upper part) or the Normotype-control avatar (lower part) and the avatar reflection in the virtual mirror. The participant has provided consent for the publication of the image. (**B**) Right-arm motion tracking: the participant’s movements were congruently mapped, in terms of space and time, on the avatar right arm. (**C**) Snapshot of physiological tools used to record SCR and HR in Study 1.

Participants observed the virtual body from 1PP through the HMD (Fig. 2A) and could control in real time the movements of their right forearm by means of a simple procedure based on the Oculus internal tracking system. In fact, we implemented a customized script in Unity able to track the position of the Right Oculus Controller that participants hold with the right hand, and to display similar movements on the avatar right forearm (see Fig. 2B) which, in turn, was programmed to rotate around a vector pointing at Oculus Controller current position.

#### Virtual scenario and experimental tasks

The virtual scenario and the tasks, of both Study 1 and Study 2, were designed using Unity Game engine software and presented through the HMD. Participants found themselves in a simple naturalistic environment with a virtual turf similar to natural grass. The only object in the scene was a mirror set in front of the participants reflecting the virtual body from the chin down (Fig. 2A). In both studies, two motor exercises, used to enhance the feeling to control the avatar movements, were implemented: (1) *motor adaptation task*, where participants were asked to follow with their real right hand the outline of a set of four geometrical figures that appeared close to their virtual right hand; (2) *motor task*, consisting in reaching a sequence of yellow transparent virtual balls by accurately moving their right arm^18,26^. Moreover, during both studies participants were required to answer different questions using a Visual Analogic Scale (VAS) ranging from 0 to 100 and built in a black panel that appeared in the virtual environment in front of the virtual body. Participants were instructed to use the right controller analog stick with their right thumb that moved the cursor along the VAS line in order to provide their responses. None of the participants reported or exhibited any difficulty in following the instructions.

Finally, two different conditions were implemented in the virtual scenario for Study 1 and Study 2 respectively. In particular, in the former, to measure participants’ perception of feeling physically in danger, a threatening event has been implemented in the virtual scenario, i.e., the ground suddenly collapsing under the avatar feet, isolating them on a small platform at 22 m above the ground (Fig. 1D). Regarding the latter, to investigate participants’ estimation of their own physical abilities, we implemented a simple task in the virtual scenario (estimated distance task), where participants could use their right thumb on the Oculus controller analog stick, for moving forward or backward the ground platform in front of them until it reached the maximum distance they thought they were able to jump over (Fig. 1E).

### Ethics declarations

The experimental protocol (CE/PROG.879) was approved by the ethics committee of the Fondazione Santa Lucia and was carried out in accordance with the ethical standards of the 2013 Declaration of Helsinki. All participants gave their written informed consent to take part in the study. Participants were compensated for their participation by receiving €12.

### Common measures

#### Embodiment illusion questionnaire

To assess the degree to which participants experienced the embodiment illusion over each avatar we presented them an 8-items questionnaire adapted from previous studies^3,5,9,11,39^. Four items referred to participant’s perception of owning the avatar body (i.e., the feeling that the virtual body is one’s own body), and four items measured the perceived agency over the virtual body (i.e., the feeling to be in control of the avatar movements) (see Table 1 for a detailed description of each item).

**Table 1:** Embodiment illusion questionnaire. Embodiment_Index = [(Q1−Q2 + Q3−Q4)/4] + [(Q5 + Q6−Q7−Q8)/4].

Q1	Body ownership	experimental	I felt as if the virtual body was my body
Q2	Body ownership	control	It seemed as if I might have more than one body
Q3	Body ownership	experimental	I felt as if the virtual body I saw was my own body
Q4	Body ownership	control	I felt as if the virtual body I saw was another person
Q5	Agency	experimental	It felt like I could control the virtual arm as if it was my own
Q6	Agency	experimental	I felt as if the movements of the virtual arm were my own
Q7	Agency	control	I felt as if the movements of the virtual arm were influencing my own movements
Q8	Agency	control	I felt as if the virtual arm was moving by itself

#### Other measures

Socio-demographic information like gender, age, religious affiliation and education were collected a week before the two studies took place.

Moreover, five questionnaires were administered to control for potential moderators:

*Subjective Omnipotence Scale (SOS) (Cronbanch’s α* = *0.89) and Adolescent Invulnerability Scale (AIS) (Cronbanch’s α* = *0.87):* two 5-point Likert scales from 1 (Strongly disagree) to 5 (Strongly agree), developed from the New Personal Fable Scale^40^, to assess, respectively, omnipotence (30 items. Subscales: Influence, Leadership, Grandiosity) and feelings of Invulnerability (20 items. Subscales: Danger Invulnerability, Psychological Invulnerability).

*Rosenberg Self Esteem Scale (RSE) (Cronbanch’s α* = *0.89)*: a 4-point Likert scale from 1 (Strongly agree) to 4 (Strongly disagree), composed of 10 items measuring Self-Esteem^41^.

*General Risk Propensity (GRiPS) (Cronbanch’s α* = *0.92)*: a 5-point Likert scale from 1 (Strongly disagree) to 5 (Strongly agree), composed of 8 items measuring people's general propensity to take risks across situations^42^.

*The Index of Spiritual Experience (INSPIRIT) (Cronbanch’s α* = *0.82)*: a 4-point response scale composed of 7 items measuring the individual’s spiritual experience independent of religion^43^.

## Study 1

The first study aimed at investigating the potential effect of embodying a supernatural agent (i.e., God) on people’s perception of physical invulnerability at both behavioural and physiological level by manipulating the virtual body that they could embody (God-avatar, Muscled-control avatar, Normotype-control avatar). We expected people in the control avatars to similarly experience the threatening event as more arousing compared to the God condition, by observing an increase in skin conductance response and a decrease in beats per minute consequently.

### Procedure

Participants were recruited via email and, after being checked for exclusion criteria, were required to carefully read and sign the informed consent. Participants were told that the purpose of the study was the validation of a set of avatars to be used in future research. As part of the cover story, they were told they were randomly assigned as raters of three (Christian God and the two control avatars) out of nine avatars from a list that was read to them. This expedient served us primarily to avoid the risk that participants might mistakenly recognize some other character with similar physical features instead of God (e.g., a saint). At the same time, it served to prevent being totally explicit and induce any possible compliance effect.

Then, participants wore the HMD through which they observed the virtual scenario and the avatar (from 1PP) to which they were assigned. They were instructed on how to use the joysticks to interact with the virtual environment. Before starting the experimental task, participants underwent a calibration phase, during which their point of view was matched with that of the avatar, and a 5-min orientation phase during which they were asked to visually explore their body by looking down at their feet and looking at themselves reflected in the virtual mirror set in front of them. During this time, they were asked also to explore the capabilities of their virtual body by performing the *motor adaptation task* (described in Common Experimental Stimuli and Setup section).

Then, the experimental phase started: participants were firstly required to perform the *motor task*, i.e., touch the virtual balls appearing next the avatar right hand. The balls appeared in positions such that participants were forced to look downward and focus on their virtual arm and body, as well as on the ground platform (which would collapse at the end of each trial). To keep the participant attentive, we stressed that the accuracy of the movement was of extreme importance for the study because on this parameter we would have based the validation of the avatars.

**Figure 3:**
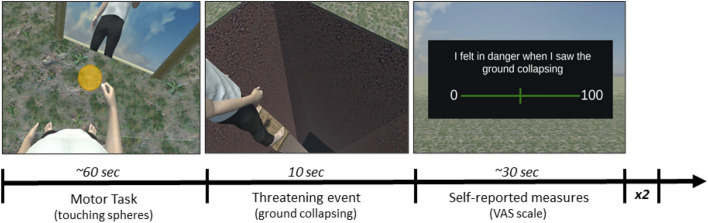
Study 1 Timeline, which included the motor task (touching the virtual spheres), the threatening event (ground collapsing), Self-reported measures.

After about 60 s from the beginning of the motor task the ground collapsed around the participants’ feet, isolating them on a small platform at 22 m above the ground. After 10 s the ground was restored, the VAS panel appeared in front of the participants who were asked to report feelings (see Emotional self-report measures below) related to the threatening event. Then, the *motor task* started again and, after about 30 s, a second collapse of the ground occurred, followed by the self-reported measures (Fig. 3). The procedure was repeated for the remaining two avatars with 15 min break at the end of each block. The order of presentation of the avatars to be embodied was counterbalanced across subjects.

At the end, participants were asked which of the 9 avatar of the list they embodied to make sure they did recognize the avatar they were in.

### Measures

#### Physiological activity

Skin Conductance Response (SCR) and heart beats per minute (BPM) were recorded in response to the threatening event onset (ground collapsing, Fig. 1D). Moreover, also heart rate deceleration (HRD) was included, since this parameter has already been associated with aversive stress in response to threats to the virtual body (e.g.,^21^).

ADInstruments PowerLab 8/35 and ML116 Galvanic Skin Response (GSR) Amplifier [providing a 75-Hz AC excitation with low constant voltage of 22 millivolt root mean square (mVrms)] devices were used to amplify the signals and specific GSR sensors consisting of two bipolar finger electrodes placed on the left hand. The signal was sampled at 1 kHz, recorded and analysed using LabChart (ADInstruments, version 7.3.8) and Ledalab, a Matlab-based software for the analysis of skin conductance data (http://www.ledalab.de/, version 3.4.9). The same devices were used to record participant’s electrocardiogram (ECG). A convenience lead II ECG signal was recorded placing the electrodes on the abdomen (DORMO pregelled electrodes 50 mm). Signals were sampled at 1 kHz and filtered using a 30-Hz low-pass filter (Fig. 2C).

#### Emotional self-report measures

After each presentation of the threatening event, participants were presented with six questions concerning their feelings, to which they answered using VAS. The questions were: (1) “I felt in danger when I saw the ground collapsing”, (2) “I felt like I could fall when the ground collapsed”, (3) “I felt anxious when the ground collapsed”, (4) “I felt relaxed when the ground collapsed”, (5) I felt safe when I saw the ground collapsing” 6) I was afraid of getting hurt when the ground collapsed”*.*

### Common preliminary analysis

#### Embodiment illusion

**Figure 4:**
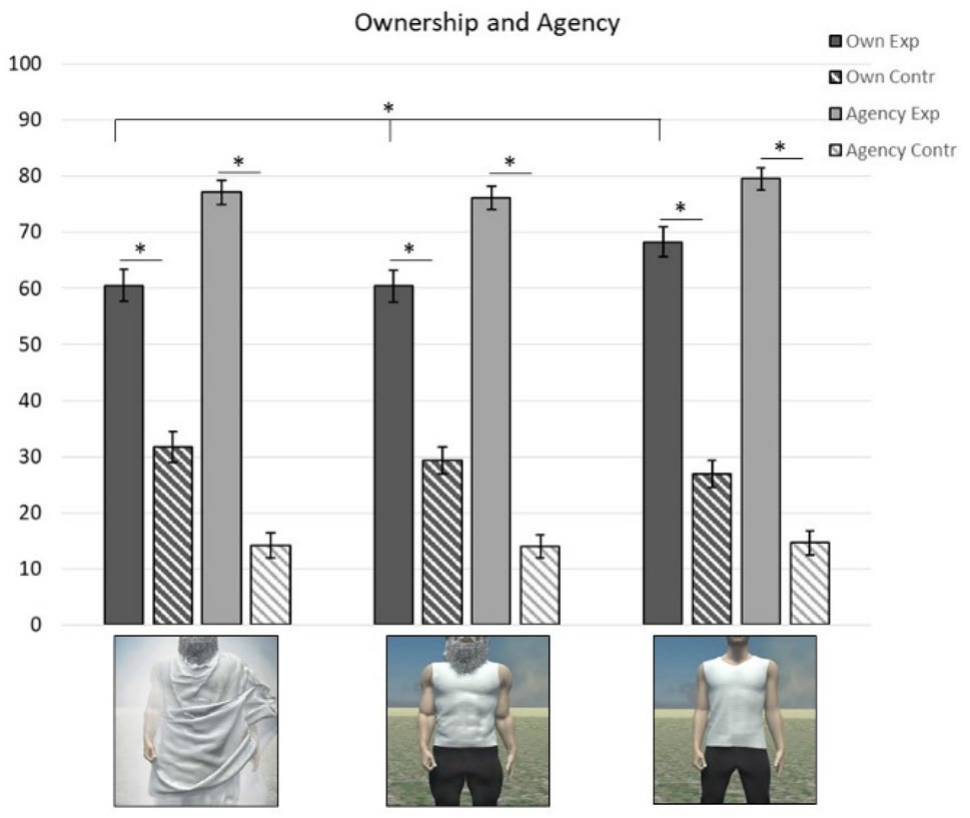
Embodiment illusion scores for each avatar: Dark bars and striped dark bars represent the mean scores of Ownership-experimental items and controls, respectively. Grey and stripped grey bars represent the mean scores of Agency-experimental items and controls, respectively.

A one-way repeated measured analysis of variance (ANOVA) was performed by using IBM SPSS Statistics^44^ to evaluate the null hypothesis that there is no difference in participants’ *Embodiment illusion* scores after embodying the three avatars *(God-avatar, Muscled-control avatar, Normotype-control avatar).* Specifically, experimental and control scores for both ownership and agency dimension were separately analysed after we excluded participants with missing recordings (N = 1), and those who did not recognize the God-avatar (N = 2). The multivariate test results indicated that there were significant differences in ownership-experimental scores across avatars, Wilks’ Lambda < 0.01, F(2,49) = 8.61, *p* < 0.01, η^2^ = 0.26. Mauchly’s test χ2 (2) = 4.37, *p* = 0.11 did not indicate any violation of sphericity. The difference between means was statistically significant, F(2, 100) = 7.46, *p* < 0.01, n2 = 0.13, again indicating significant differences in *Ownership-experimental* score. Follow up comparison indicated that the participants’ *Ownership-experimental* score after embodying the Normotype-control avatar (M = 68.28, SE = 2.70) was higher compared to both the God-avatar (M = 60.55, SE = 2.92) and the Muscled-control avatar (M = 60.40, SE = 2.85). No significant differences were found comparing *Ownership-control, Agency-experimental, Agency-control* scores (all ps > 0.13 e all Fs < 2.12) (Fig. 4).

To make sure that any result was attributed to the avatar manipulation, and not to the embodiment illusion differences, we included the embodiment illusion total score (*Embodiment Index*) as a continuous predictor in all our analyses (Study 1 and Study 2) (see Table 1 for the scoring formula).

### Analysis and results

#### Skin conductance responses

Data pre-processing was carried out using Matlab (The MathWorks, Natick, MA, version R2019b) and the Matlab-based toolbox Ledalab (Leipzig, Germany; www.ledalab.de, version 3.4.9). Continuous decomposition analysis (CDA^45^,) was performed to separate phasic components from tonic activity based on standard deconvolution. Skin Conductance Response (SCR) was analysed using the mean amplitude of the phasic SCR within a window of 10 s after the threatening event onset with a minimum response of 0.01 μS. To overcome computational limits, data were down sampled to 10 Hz.

First, we analysed data relative to both falls: we excluded eight participants with missing or invalid recordings and two participants who did not recognize the God-avatar (total N = 44).

Data analysis was performed using R software^46^ (version 4.1.3) via RStudio^47^ (version 2022.02.0). We performed a multilevel mixed linear regression analysis (LMM or “mixed-effects models^48,49^”;) through the package *lme4* Version 1.1–5^50^ with *CDA.SCR* as continuous dependent variable, the categorical variable *Avatar (God-avatar, Muscled-control avatar, Normotype-control avatar)*, the continuous variable *Embodiment index* (centered to the mean) and their interaction as fixed effects. As suggested by Barr^51^, we built the random part with the maximal structure allowed by our data without incurring in convergence issues, which resulted in the random intercept over participants.

Statistical significance of fixed effects was determined using type III Wald F test with Kenward–Roger degrees of freedom^52^ and the *Anova* function from *car* package. Post hoc pairwise comparisons (Tukey corrected) were performed using least squares contrasts (lsc), as employed in R’s *lsmeans* package.

In order to select the variables to include in the model as covariates, we set a null model with *CDA.SCR* as the dependent variable, participants’ score on the questionnaires and their demographic information as not-interacting fixed effects, i.e., *SOS (subscale grandiosity), AIS (subscale danger), RSE, GRiPS, INSPIRIT, Age, Gender, Education, Religion, Randomization*. As for the random structure, we set the random intercept over participants^53^. This procedure for selecting covariates was performed for all the analyses. Here, no significant effects were found, thus no covariates were added to this model.

The analysis revealed a significant main effect of *Avatar* (F(2,219.68) = 3.19, *p* = 0.04).

**Figure 5:**
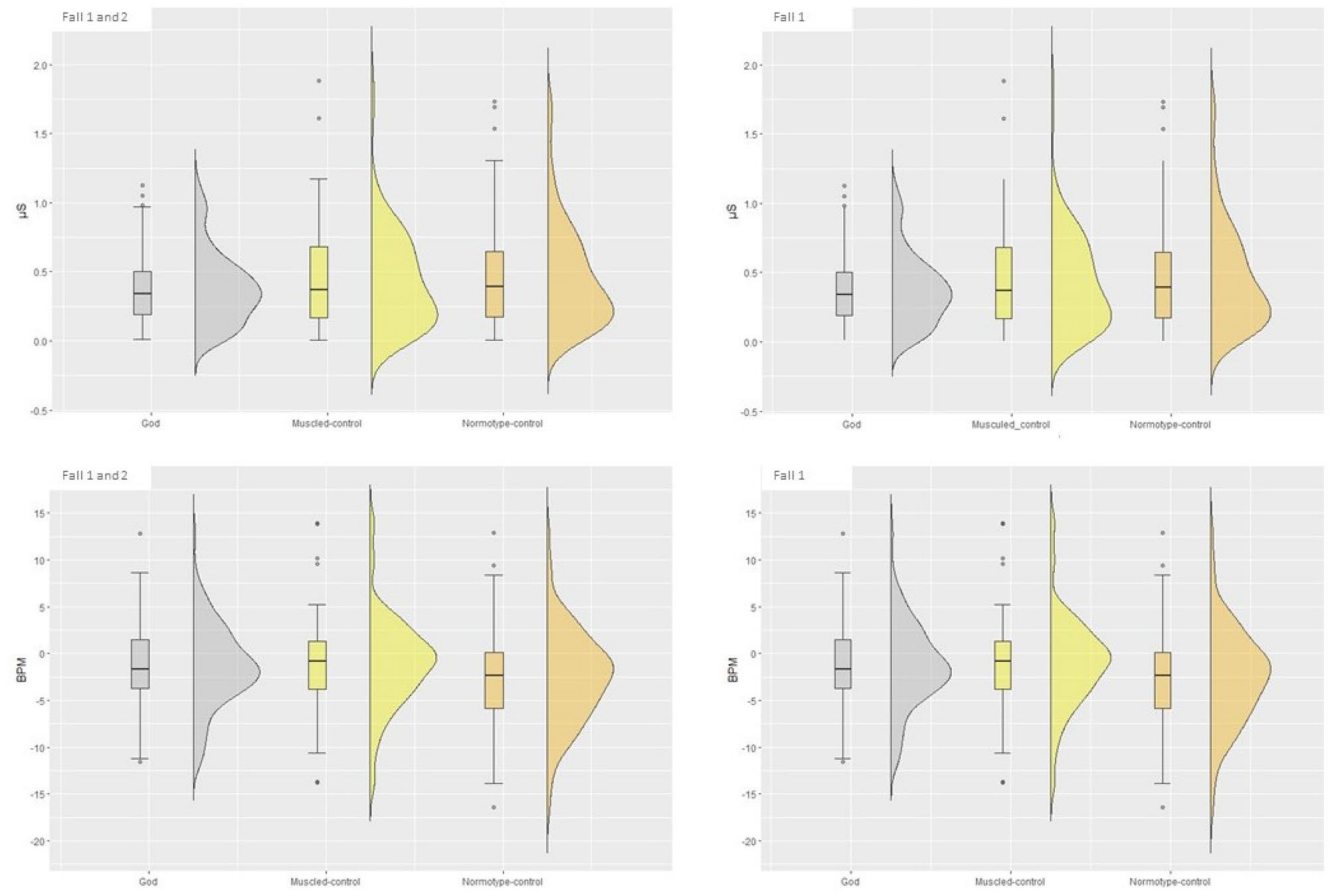
Physiological Measures: The box and violin plot show SCR (Upper part) and HRD (Lower part) recordings as a function of the Avatar embodied (God-avatar, Muscled-control avatar, Normotype-control avatar).

Specifically, participants presented a lower SCR increase during the threatening event while they were embodying the God-avatar, (M = 0.37 μS, SE = 0.05) compared to the Normotype-control avatar (M = 0.46 μS, SE = 0.05; t(222) = -2.58, *p* = 0.03, Cohen's d = -0.41, 95% CI [-0.72, -0.09]). No other significant main effects or interactions were found (Fig. 5).

We then analysed the first fall for each avatar, but no significant results emerged (all ps > 0.6 and all Fs < 2.89) (Fig. 5).

#### Heart rate

Our dependent variables consisted of beats per minute (*BPM*) during the event-related time window, and heart rate deceleration (*HRD*), a value obtained by subtracting BPM during the baseline period (i.e., 10 s before the collapse) from the BPM during the event-related time window. For each dependent variable, we performed a multilevel mixed linear regression analysis with the categorical variable *Avatar,* the continuous variable *Embodiment index* (centered to the mean) and their interaction as fixed effects.

As at the previous stage, we excluded five participants with missing or invalid recordings for both falls and two participants who did not recognize the God-avatar. Analysis on both falls (total N = 47) was performed.

Following the procedure described above, no covariates were selected to be included in the HRD model, whereas *Subjective omnipotence (subscale grandiosity)*, *Rosenberg self-esteem* and *Gender* were added in the BPM model.

The analysis on HRD revealed a significant main effect of *Avatar* [F(2, 233.86) = 4.61, *p* = 0.01]. HRD was observed during all three conditions. However, participants’ heart rate decelerated significantly less during the threatening event while they were embodying Muscled-control avatar (M = −1.09 BPM, SE = 0.59) than when they were embodying the Normotype-control avatar (M = −2.66 BPM, SE = 0.59; t(236) = 2.87, *p* = 0.01, Cohen's d = 0.43, 95% CI [0.13, 0.72]). No significant difference was found between the God-avatar (M = −1.41 BPM, SE = 0.59) and Normotype-control avatar (t(236) = 2.27, *p* = 0.06), as well as between the God-avatar and Muscled-control avatar (t(230) = −0.61, *p* = 0.82) (Fig. 5).

The BPM analysis revealed no significant main effects or interaction (all ps > 0.29 and all Fs < 1.22).

We also analysed the first fall for each avatar (total N = 47). Analysis on HRD revealed a main effect of *Avatar* (F(2, 92.92) = 7.01, *p* < 0.01]. Post-hoc analysis revealed that participants’ heart rate decelerated significantly less when participants were embodying the God-avatar (M = -1.60 BPM, SE = 0.73) than when they were embodying the Normotype-control avatar (M = -3.95 BPM, SE = 0.74; t(93.6) = 3.10, *p* = 0.01, Cohen's d = 0.64, 95% CI [0.22, 1.06). Moreover, participants’ heart rate decelerated significantly less also in the Muscled-control avatar (M = -1.40 BPM, SE = 0.73) compared to the Normotype-control avatar (M = -3.95 BPM, SE = 0.74; t(93.9) = 3.35, *p* < 0.01, Cohen's d = 0.70, 95% CI [0.28, 1.12]). No difference between the God-avatar and Muscled-control avatar was found (t(91.3) = -0.26, *p* = 0.96) (Fig. 5).

Again, the BPM analysis revealed no significant main effects or interaction (all ps > 0.45 and all Fs < 0.82).

#### Emotional self-report measures

Item scores from Self-report measures was summed in an index, which we will refer to as the *Explicit Index*.

To test the effect of the avatar and the level of the embodiment on participants’ response to the self-reported questions, we performed a multilevel linear regression relative to both falls with *Explicit Index* as the continuous dependent variable and *Avatar*, *Embodiment Index* and their interaction as fixed effects. As for the random structure we set the random intercept over participants. Following the procedure described above, no covariates were added to the model. We found a significant Avatar*Embodiment index interaction [F(2, 254.99) = 6.10, *p* < 0.01]. We excluded a participant with missing recordings, and two participants who did not recognize the God-avatar to first analyse data from both falls (total N = 51).

Simple slopes analyses revealed that the more the participants embodied the Muscled-control avatar, the less they stated to have experienced the event as threatening (B = −0.37, SE = 0.14, t(297) = -2.67, 95% CI [−0.64, −0.10], *p* < 0.01).

Just considering the first fall per avatar, no significant effects resulted.

## Study 2

To test the potential effect of embodying a supernatural agent (i.e., God) on people’s perception of their own physical abilities, we recorded participants’ estimates of the maximum distance they thought they were able to jump over.

We expected that participants would estimate they could jump farther while embodying the God-avatar compared to both Muscled-control and Normotype-control avatars. In fact, participants were supposed to partially transfer on themselves God’s ultimate power via top-down processes, to the extent that they could alter the perception of their own limits. However, because of its conspicuous muscle mass, we expect also the Muscled-control control avatar to exert an effect, although smaller than that exerted by the God-avatar.

### Procedure

The same cover story of Study 1 was told to hide the real experimental purpose and to avoid that participants intentionally responded accordingly with the hypotheses.

**Figure 6:**
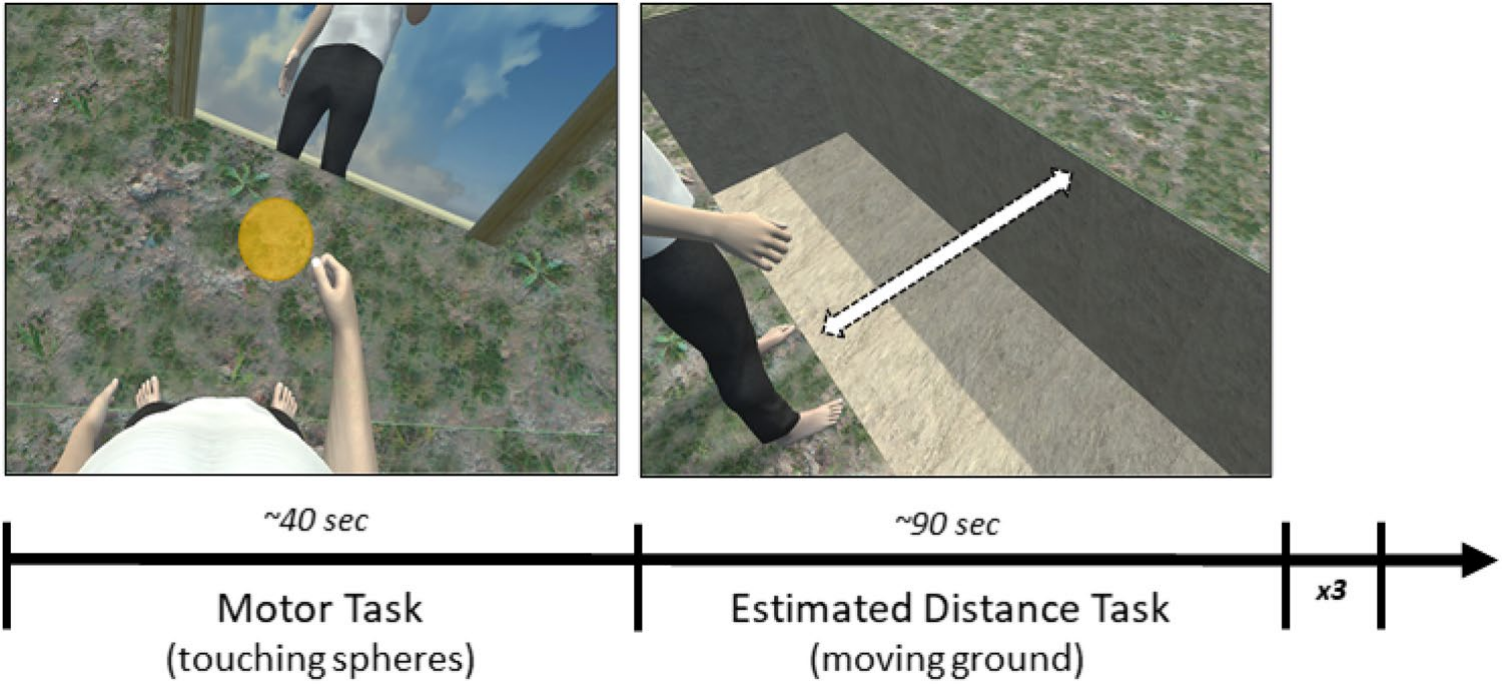
Study 2 Timeline including the Motor Task (touching the virtual spheres), the Estimated Distance Task (moving forward or backward the ground platform in front of them until it reached the maximum distance that participants thought they were able to jump over).

Once they wore the HMD, before starting the experimental task, participants underwent to a calibration phase, during which their point of view was matched with the avatar one, and a 5-min familiarization phase with the first of the three avatars was performed. The order of presentation of the three avatars was randomized. Participants performed the *motor adaptation task* (described in Common Experimental Stimuli and Setup section) and then moved the ground platform in front of their feet forward until it reached the maximum distance they thought they were able to jump over. A chasm with three different possible depths opened wide when participants began moving the platform. A total of 27 trials divided in three blocks were completed. At the end of each block participants performed *motor tasks* of twenty seconds each in order to maintain a strong embodiment illusion (Fig. 6).

The procedure was repeated for the remaining two avatars with 15 min break at the end of each block. The order of presentation of the blocks was counterbalanced across subjects.

At the end, participants were asked which of the 9 avatar of the list they embodied to make sure they did recognize the avatar they were in.

### Measures

#### Estimated distance

We recorded the distance between the platform starting point and the point where the participant stopped the platform. Each participant completed a total of 27 trials per avatar (9 trials per depth level), randomly presented.

### Analysis and results

#### Estimated distance

At the data preparation stage, we coded *Distance* values equal to zero as missing values, as these indicated that the participant mistakenly pressed the button and skipped the trial.

We excluded a participant who could not complete the task and two participants who did not recognize the God-avatar (total N = 51).

We performed a multilevel mixed linear regression analysis with *Distance* as the continuous dependent variable, the categorical variables *Avatar* (*God-avatar, Muscled-control avatar, Normotype-control avatar)*, and *Depth (low, medium, high)*, and the continuous variable *Embodiment index* (centered to the mean) and their respective interactions as fixed effects. As for the random structure, we considered the random intercept over participants. We used the same method as in Study 1 to select the covariates to be included in the model, which resulted in only Gender.

The analysis revealed a significant main effect of Avatar [F(2,3932.5) = 22.66, *p* < 0.01], Depth [F(2,3932) = 68.36.23, *p* < 0.01] and Embodiment Index [F(1,3945.2) = 4.89, *p* = 0.03]. No significant interactions were found.

**Figure 7:**
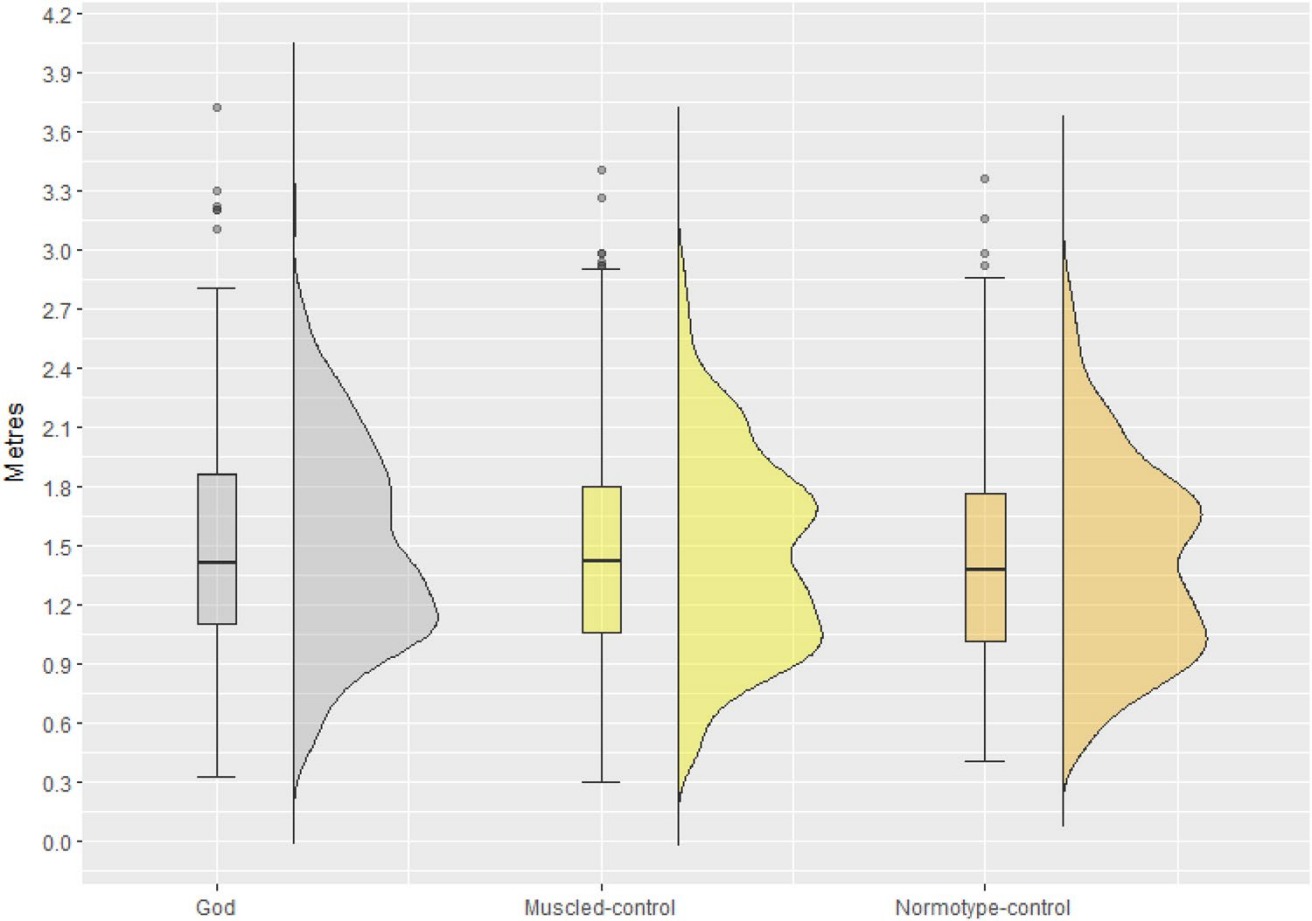
Estimated Distance: The box and violin plots show all the 27 evaluations performed by each participant per condition (God-avatar, Muscled-control avatar, Normotype-control avatar).

Post-hoc analysis showed that participants estimated they could jump farther when participants were embodying the God-avatar (M = 1.49 m, SE = 0.07) than when they were embodying the Muscled-control avatar (M = 1.46 m, SE = 0.07; t(3932) = 4.90, *p* < 0.01, Cohen's d = 0.19, 95% CI [0.11, 0.27]) and the Normotype-control avatar (M = 1.41 m, SE = 0.07; t(3934) = 12.35, *p* < 0.01, Cohen's d = 0.49, 95% CI [0.42, 0.57]). Furthermore, when participants were embodying the Muscled-control avatar they estimated they could jump farther compared to the Normotype-control avatar (t(3934) = 7.60, *p* < 0.01, Cohen's d = 0.30, 95% CI [0.23, 0.38]) (Fig. 7).

With regard to *Depth*, post hoc analysis showed that participants estimated they could jump farther at low depth (M = 1.54 m, SE = 0.07) than at medium depth (M = 1.42 m, SE = 0.07; t(3932) = 19.57, *p* < 0.01, Cohen's d = 0.76, 95% CI [0.68, 0.84]). At the same time, at low depth they estimated they could jump farther than at high depth (M = 1.41 m, SE = 0.07; t(3932) = 20.99, *p* < 0.01, Cohen's d = 0.82, 95% CI [0.74, 0.90]) No difference was found between medium and high depth (t(3932) = 1.47, *p* = 0.31).

Finally, the *Embodiment Index* was found to significantly predict the evaluation. Specifically, the more participants embodied the avatar, the farther they thought to be able to jump (B = 0.01, *p* < 0.03).

## General discussion

Previous studies have demonstrated that people immersed in VR feel the illusion of owning virtual bodies as if they were their own, even when these virtual bodies differ from their real one in conspicuous characteristics like sex^21,54^, age^26,29,30^, skin colour^17,25,28,55,56^, size^14,57,58^. Moreover, studies indicate that this illusion works also when people embody virtual bodies resembling certain specific individuals (e.g., Einstein, Freud, Lenin. See^30,31,59^. Importantly, in these cases participants appeared to adhere to the new identities by behaving accordingly with the hallmarks of the characters they were embodying. Our results support and expand these findings by suggesting that people can embody also the physical representation of an abstract entity (i.e., the Christian God).

Overall, we found that the embodiment illusion was elicited by all the three avatars, with a slight difference in favour of the Normotype-control avatar just for Ownership-experimental items. This outcome was not surprising and it is probably due to the fact that the Normotype-control body was more akin to the physical appearance of our participants, who did not present a pronounced fitness level. It may be worth noting that literature is not consistent on this point. While some studies found no difference in ownership ratings among conditions (e.g.,^17^), others did (e.g.,^5,12,54^), supporting the thesis that the avatar appearance might enhance or inhibit the embodiment illusion^39^. Importantly, ratings in the God-avatar and in the Muscled-control avatar were similar, supporting the idea that the difference with the Normotype-control avatar depended only on the avatar body mass and not on the participants’ possible difficulty in feeling an abstract entity as their own the body. To control if this difference could have affected our results, we included an index of the embodiment illusion scores in our analyses. Interestingly, although minimal and extremely simple, our motion capture system proved effective in eliciting the embodiment illusion, as reported by our participants which stated that they perceived full control over the actions performed by the virtual body arm. Since it does not involve any wearable suit, a system such as the one presented here might considerably simplify, both in terms of time and resources, the use of motion capture technology.

We found that embodying the anthropomorphic representation of the Christian God seem to lead participants to reshape their own personal representation accordingly with its attributes. Specifically, virtually being an entity with absolute power seems to affect physiological reaction to a threatening event (Study 1) and explicit evaluation of one’s own physical abilities (Study 2).

In fact, partially in line with our hypothesis, results of Study 1 indicate that embodying the God-avatar lead people to perceive the aversive event virtually compromising their physical safety as less threatening, as demonstrated by a lower SCR increase, but only when confronted with the embodiment of the Normotype-control avatar and when both falls were analysed together. In the same vein, studies indicate that events perceived as threatening may lead to an increase in SCR and a lower heart rate (e.g.,^4,8,13,60–64^).

Conversely, no differences in BPM were found. Nonetheless, the analysis of HRD, a parameter that has also been measured as reflecting freezing reactions in response to aversive events^21,54,63–65^ revealed that participants’ heart rate decelerated in all the three conditions. However, when participants were embodying the God-avatar and the Muscled-control avatar the HRD was significantly lower in contrast to the Normotype-control avatar, suggesting a weaker freezing reaction. Interestingly, while embodying the God-avatar seemed to partially affect participants’ physiological responses, no effect was observed in explicit ratings whereby participants rated the event as equally threatening in all the three conditions. We speculate that complex factors like social desirability might have influenced explicit responses that are typically controllable, intended and made with awareness^66^. However, it might also possible that our measure was not sensitive enough to grasp a difference in the event evaluation or, simply, that the avatar manipulation does not work at the explicit level, while eliciting changes at the unconscious and uncontrollable level. It is worth noting that the results related to physiological responses results were only partially in line with our expectations: if, on the one hand, a difference in SCR and HRD emerged between the God-avatar and the Normotype-control avatar, on the other hand we also expected a similar outcome between the God-avatar and the Muscled-control avatar, which however did not occur. In fact, we expected that none of the two control human avatars would have affected participants’ physiological responses as none of their physical traits could have been useful to escape the specific threatening event that we presented them. However, results in HRD seem not to support this hypothesis, and having a different virtual body mass may also affect the sense of physical invulnerability. Even if the Muscled-control avatar did enhance the sense of invulnerability, we speculate that the sense of invulnerability that people transfer on themselves from God’s omnipotence might qualitatively differ from that derived from embodying a mere muscular body. Indeed, were the effects merely associated to muscle mass, we would have found more consistent findings in favour of the Muscled-control avatar. In fact, although the God-avatar and Muscled-control avatar had the very same body mass, the Muscled-control avatar was rated as significantly more muscular (see Supplementary materials). Tellingly, further evidence supporting this explanation comes from Study 2, in which we found that the sense of power deriving from the embodiment of the God-avatar affected participants’ evaluation of their own physical abilities. Consistently with our expectations, participants estimated they could jump farther in the God-avatar condition compared to both the Muscled-control and the Normotype-control avatars. In addition, a significant effect was found also between the Muscled-control avatar and the Normotype-control avatar, leading us to the conclusion that body mass played a role in the physical ability evaluation. Once again, we observed that body mass was not the only factor driving the effects. In fact, even though the Muscled-control avatar was rated as more muscular than the God-avatar (see Supplementary materials), participants estimated they could jump farther in this latter condition. In line with Study 1, we can therefore argue that embodying a model who is specifically linked to absolute power leads to more prominent changes in one’s personal agency, rather than merely having a muscular body linked to physical strength. Noteworthy is the fact that the averages in the distance estimation task were close among avatars. This outcome supports the thesis that the illusion worked without participants’ consciousness and there was no compliance effect. In Study 2 it is interesting to note that the effect of embodying different avatars did not interact with the chasm depth. Future developments may further test this point, for example, by increasing the number of depth levels and making the measure more sensitive.

To sum up, results of Study 1 and Study 2 suggest that the God-avatar may exert an influence on people’s perception of their own limits and capabilities. However, further investigations are required to clearly determine the extent to which the transformative effect works. In addition, we found evidence that embodying a certain character to whom people ascribe an absolute power or an avatar with the very same physical attributes to whom people may associate physical strength leads to different outcomes. This is a crucial point for our research field. Indeed, researchers who aim at taking advantage of the body ownership illusion to generate specific changes should consider that embodiment experiences in avatars resembling certain role models could be more effective in determining changes. For instance, extending Banakou and colleagues’ findings^30^, embodying the Albert Einstein avatar, who is the prototype of the super intelligent man, might be more effective in enhancing executive functioning compared to an avatar representing a generic scientist, i.e., the category to which Einstein belongs. Similarly, embodying a prototype of the inventor category, e.g., Leonardo da Vinci, rather than a generic inventor, might better support people in generating original ideas^67^. This argument can be applied to several contexts: for example, in the field of clinical rehabilitation we are witnessing a growing interest in the use of the body ownership illusion in pain modulation (for a review see^68^). After determining that avatar features modulate pain thresholds^69–72^, some authors are wondering on the possible use of embodiment illusion to create a pain-free representation of the body^68^. In the light of our findings suggesting that the God-avatar might enhance one’s sense of physical invulnerability, future developments might answer this question by testing if the God-avatar can modulate real aversive events, like painful stimulations, and comparing its effectiveness with that of avatars varying in basic physical attributes.

Although supporting and extending in a promising way research findings related to the *Proteus effect*, the present results come with some limitations: first, our findings cannot be generalized outside the Christian-Catholic context, and, more specifically, the Italian one. In fact, it is reasonable to think that the God-avatar that we employed could work only if participants correctly recognize the entity and activate those specific traits and attributes associated to it. In this vein, people not born and raised in a Christian cultural context might not have the same ease of recognition. Furthermore, even within the same Christian cultural context there are some differences, e.g., in the exhibition of sacred images, which might help participants to recognize the Christian God-avatar in its anthropomorphic form. In addition, several works suggest that people have an anthropomorphic representation of God’s mind, which involves attributing to it human emotions and analytic abilities^73^. To the best of our knowledge, no studies have explored the unique contribution of cultures in ascribing certain traits to God, however, it is likely that differences in cultures result in differences in God’s mind representation. This point becomes crucial in the investigation of other dimensions potentially affected by the God-avatar embodiment, e.g., the moral sphere, where the personality traits people attribute to God could be expected to considerably direct any transformative effect. Considering recent advances exploring the influence of body ownership illusion on moral cognition^20^, future investigations adopting the God-avatar might here find a fertile ground.

Another important issue concerns people’s religious orientation. Assuming that Italian culture is deeply rooted in the Catholic-Christian religion, we found reasonable to recruit Catholic-Christian participants, but also agnostics and atheists. In fact, we assumed that recognizing the God-avatar would activate those characteristics associated to it and to which people are used to be exposed to, regardless of whether they believe it or not. However, it is reasonable to hypothesize that the stronger the belief in God, the stronger the effect that follows. In the present work, though, neither the religious orientation nor spiritual level proved to have an effect on our dependent variables. We recommend future developments to carefully consider these issues, especially in the case of between-subjects design experiments, where, if not matched, participants’ characteristics might considerably affect the results. In this regard, deciding for a within-subject design allowed us to deal with interindividual variability and, consequently, to have a strong statistical power^48^. However, this choice might also represent a limitation, at least in Study 1, where multiple exposures to the threatening event might lead to a habituation effect (e.g.,^74^). To deal with this potential issue we carefully randomized the order of the avatars to which participants were assigned. Further confirmations are required, and future insight may benefit from additional physiological parameters of freezing behaviour, such as arterial pressure^75^, muscle tonus^76^ and body temperature^77^.

With regard to Study 2, it could be intriguing to explore if embodying an omnipotent avatar may actually enhance one’s physical strength in tasks in which the participant is asked to perform rather than just to evaluate. For instance, researchers might use specific tools, such as hand grip dynamometers, to measure force and grip pressure applied by participants while embodying God or other avatars.

Finally, the major challenge remains that of devising tasks assessing omnipotence in its reflections. Although it is impossible to measure this concept directly in practice, the sense of omnipotence can be reflected by actions or attitudes. In the present work, we measured changes in perception of physical capabilities and limits, but new tasks reflecting other facets of omnipotence might be helpful in drawing more straightforward conclusions.

## Supplementary Information

Supplementary Information.

## Data Availability

The datasets generated and analysed during the current study and supplementary materials are available on the Open Science Framework repository (https://osf.io/xc28t/).
