# Supplementary material for: Embodying the avatar of an omnipotent agent modulates the perception of one’s own abilities and enhances feelings of invulnerability
Source: Sci Rep. 2022 Dec 14;12:21585. doi: 10.1038/s41598-022-26016-1 (PMC9751071; doi:10.1038/s41598-022-26016-1)
Supplement: Supplementary file 1 — Supplementary Information. [file 41598_2022_26016_MOESM1_ESM.docx]

**Supplementary materials


Pilot Study 1 – Avatar Validation**

**Participants**

We recruited 259 respondents (255 were Italians, 169 females; age: M=31.10, SD ± 10.92, min=17, max=65), who have fully completed the survey. One respondent had an elementary school diploma (0.4%), 2 (0.8%) had a middle school diploma, 82 (31.7%) a high school diploma, 138 (53.3%) a bachelor or master’s degree, and 36 (13.9%) a doctoral degree or a master post-lauream. Among them, Catholic-Christian respondents were 124 (47.9%), Protestant Christians were 1 (0.4%), Orthodox Christians were 3 (1.2%), Atheists were 68 (26.3%), Agnostics were 53 (20.5%), Buddhists 2 (0.7%), and 8 respondents (3.1%) reported that they did not belong to any of the listed categories. On a 5-point scale ranging from 1 (“Not at all”) and 5 (“Strong”) measuring how strongly respondents considered themselves religious, respondents’ mean was 2.21 (SD= 1.11). About how often they spend time on religious practices, 158 respondents (61%) reported “once per year or less”, 52 (20.1%) reported “Once per month to several times per year”, 26 (10%) reported “Once per week to several times per month”, and 23 (8.9%) reported “Several times per day to several times per week”.

**Materials and Methods**

*Stimuli*To design the avatar of God that we used in this first pilot study (Fig. 1A), we based on works by Meier et al. ^1^, Jackson et al. ^2^ and Roberts et al. ^3^. The Muscled-control avatar and the Normal-control avatar were the same described in the manuscript.

**Procedure**
Respondents were asked to fill in an online survey in which they were presented with the pictures of the three avatars and asked to rate on a VAS from 0 to 100 how muscular the avatars looked. Then, they were again presented with the picture of the avatar of God and asked to write what or who they thought was being shown. Socio-demographic information like gender, age, religious affiliation, and education were collected.

**Analysis**Data analysis was performed with IBM SPSS Statistics ^4^.

*Avatar recognition*

We excluded respondents who did not fully complete the survey, who were not Italians, and were not Catholic-Christians, agnostics, or atheists, that is, our targeted groups for both studies. The final sample consisted of 241 respondents. We analysed the frequency of responses (Fig. 2A).

*Avatar muscularity*

We excluded respondents who did not answer the three questions on avatars’ body muscularity (the final sample consisted of 260 respondents).

A one-way repeated measured analysis of variance (ANOVA) was conducted to evaluate the null hypothesis that there is no difference in participants’ ratings of God-avatar, Muscled-control avatar, and Normal-control avatar muscularity. The multivariate test results indicate that there are significant differences in muscularity ratings across avatars, Wilks’ Lambda=.20, F(2,258)=532.07, p<.01, n2 = .85. Mauchly’s test is significant (p<0.1). Since Greenhouse-Geisser epsilon had a value of 0.93, we rely on Huynh-Feldt adjustment to interpret the univariate results. The Huynh-Feldt univariate test result is statistically significant, F(1.87, 485.0)=631.90, p<.01, η^2^ = .71, again indicating significant differences in muscularity ratings. Follow up comparison indicated that each pairwise difference was significant (p<.01). Respondents rated Muscled-control avatar (M=79.59, SD=0.93) as more muscular than the God-avatar (M=77.48, SD=1.03) and Normal-control avatar (M=42.62, SD=1.11).

**Result Discussion**

The percentage of respondents who correctly recognized God in its anthropomorphic form was low (18.3%) and did not allow us to use this first version of the avatar in our studies. In fact, it seemed it better represents Zeus or Jupiter (26.6%) and the percentage of answers did not differ much from that given for the more general "divinity” and “Greek/Roman divinity" categories.

Despite the fact that the God-avatar and the Muscled-control avatar had the very same physical size, respondents rated the Muscled-control avatar as significantly more muscular.

**Pilot Study 2 -** **Avatar Validation**

**Participants**

We recruited a new sample of 184 respondents (183 were Italians, 121 females; age: M=39.03, SD ± 16.07, min=18, max=90), who have fully completed the survey. Nine respondents (4.9%) had a middle school diploma, 85 (46.2%) a high school diploma, 64 (34.8%) a bachelor or master’s degree, and 26 (14.1%) a doctoral degree or a master post-lauream. Among them, Catholic-Christian respondents were 105 (57.1%), Protestant Christians were 3 (1.6%), Atheists were 38 (20.7%), Agnostics were 27 (14.7%), 1 respondent was Jewish (0.5%), and 10 respondents (5.4%) reported that they did not belong to any of the listed categories. On a 5-point scale ranging from 1 (“Not at all”) and 5 (“Strong”) measuring how strongly respondents considered themselves religious, respondents’ mean was 2.40 (SD= 1.12). About how often they spend time on religious practices, 97 respondents (52.7%) reported “once per year or less”, 38 (20.7%) reported “Once per month to several times per year”, 25 (13.6%) reported “Once per week to several times per month”, and 24 (13%) reported “Several times per day to several times per week”.

**Materials and Methods**

*Stimuli*The version of the God-avatar was used in this second pilot study is displayed in Fig. 1B. The Muscled-control avatar and the Normal-control avatar were the same described in the manuscript.  **Procedure**

We used the same procedure as in Pilot Study 1.

**Analysis**

We performed the same analyses as in Pilot Study 1, with the same exclusion criteria.

*Avatar recognition*

The final sample consisted of 165 respondents. We analysed the frequency of responses (see Fig. 2B).

*Avatar muscularity*

The final sample consisted of 181 respondents.

A one-way repeated measured analysis of variance (ANOVA) was conducted to evaluate the null hypothesis that there is no difference in participants’ ratings of God-avatar, Muscled-control avatar, and Normal-control avatar muscularity. The multivariate test results indicate that there are significant differences in muscularity ratings across avatars, Wilks’ Lambda=.22, F(=2, 179)= 322.53, p<.01, η^2^ = .78. Mauchly’s test is significant (p<0.1). Since Greenhouse-Geisser epsilon had a value of .86, we rely on Huynh-Feldt adjustment to interpret the univariate results. The Huynh-Feldt univariate test result is statistically significant, F(1.74, 313.21)=292.90, p<.01, n2 = .62, again indicating significant differences in muscularity ratings. Follow up comparison indicated that each pairwise difference was significant p<.01. Respondents rated Muscled-control avatar (M=80.46, SD=1.19) as more muscular than God-avatar (M=69.01, SD=1.46) and Normal-control avatar (M=43.87, SD=1.40).

**Result Discussion**

This new version of the avatar was much more in line with the representation of God that Christian Catholic may have. Although the percentage of respondents who correctly recognized God in its anthropomorphic form did not reach extremely high scores, it should be noted that this score is much higher than the one obtained in Pilot Study 1 and that no other figure or character achieves comparable scores in Pilot Study 2. Furthermore, this version of the God-avatar appeared not to be particularly mistaken for Zeus or Jupiter, while evoking other characters still belonging to Christianity (e.g., “Jesus”, “Saint Peter”, “Moses”, “Saint” responses, which obtained comparable scores to those of the Greek and Roman God).

It is worth noting that people might be hesitant to admit having an anthropomorphic God concept because it might appear juvenile ^5^. Moreover, in our survey respondents were asked to answer an open-ended question, that might have led them to answer using more socially acceptable labels (e.g., “Jesus”, “Moses”, characters to whom it is accepted to attribute a physical body). Considering these points and the fact we attempted to design the avatar of an abstract figure -unknowable by definition- we interpreted the fact that “God” answer was the most frequently given, with a high difference in frequency from the other proposals, as a very positive outcome.

As in Pilot Study 1, despite the fact the God-avatar and the Muscled-control avatar had the very same physical size, respondents rated the Muscled-control avatar as significantly more muscular. This result was particularly important to interpret data of Study 2.

**References**

1. Meier, B. P., Hauser, D. J., Robinson, M. D., Friesen, C. K. & Schjeldahl, K. What’s" up" with God? Vertical space as a representation of the divine. *J Pers Soc Psychol* **93**, 699 (2007).

2. Jackson, J. C., Hester, N. & Gray, K. The faces of God in America: Revealing religious diversity across people and politics. *PLoS One* **13**, e0198745 (2018).

3. Roberts, S. O. *et al.* God as a White man: A psychological barrier to conceptualizing Black people and women as leadership worthy. *Journal of Personality and Social Psychology* **119**, 1290 (2020).

4. IBM Corp., S. IBM Corp. Released 2013. IBM SPSS Statistics for Windows, Version 22.0. Armonk, NY: IBM Corp. *Google Search* (2013).

5. Barrett, J. L. & Keil, F. C. Conceptualizing a nonnatural entity: Anthropomorphism in God concepts. *Cogn Psychol* **31**, 219–247 (1996).


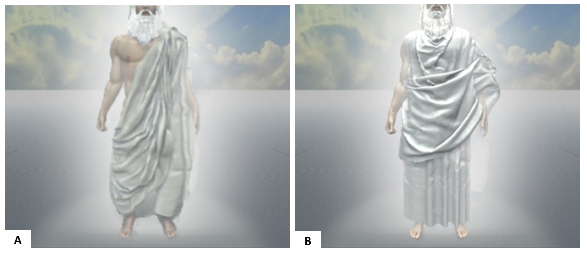


Fig. 1 A) God-avatar of Pilot Study 1; B) God-avatar of Pilot Study 2


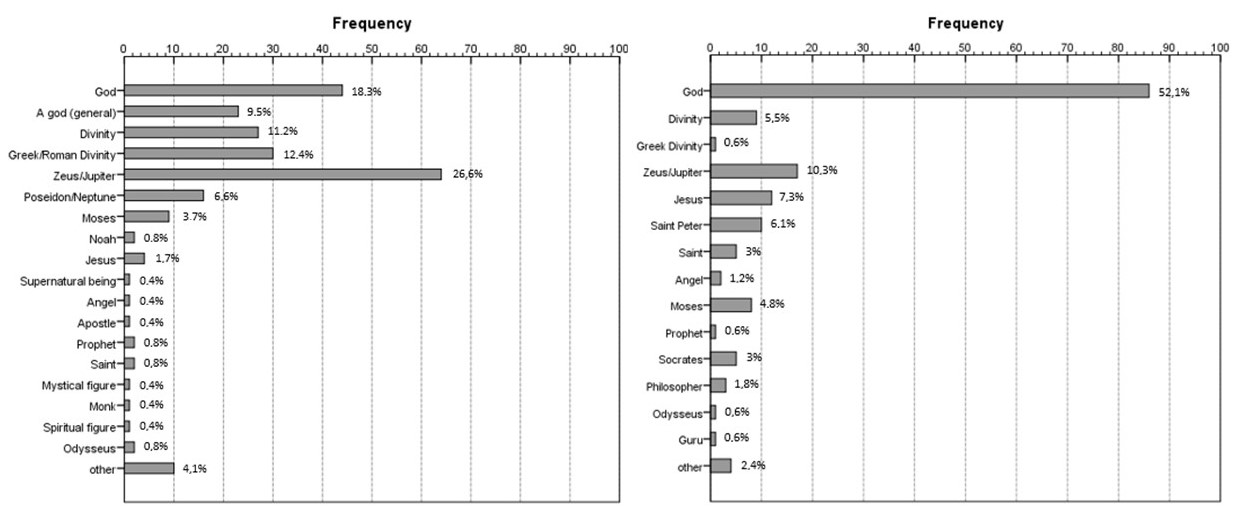


Fig. 2 Avatar recognition (frequency and percentage). Results of Pilot Study 1; B) Results of Pilot Study 2
